# A Combined four-mRNA Signature Associated with Lymphatic Metastasis for Prognosis of Colorectal Cancer

**DOI:** 10.7150/jca.38796

**Published:** 2020-02-03

**Authors:** Xueping Li, Qiang Zhang, Lan Zhao, Longyang Jiang, Aoshuang Qi, Qian Wei, Xinyue Song, Lin Wang, Liwen Zhang, Yanyun Zhao, Xuemei Lv, Minjie Wei, Lin Zhao

**Affiliations:** 1 Department of Pharmacology, School of Pharmacy, China Medical University, No.77 Puhe Road, Shenyang North New Area, Shenyang City, 110122, Liaoning, China.; 2 Liaoning Engineering Technology Research Center, China Medical University, No.77 Puhe Road, Shenyang North New Area, Shenyang City, 110122, Liaoning, China.

**Keywords:** biomarker, lymph node metastasis, colorectal cancer, mRNA, prognosis

## Abstract

**Background**: Colorectal cancer (CRC) is one of the most common malignant tumors in the world. Lymph node metastasis (LNM) is a common mode of metastasis of CRC. However, the combined mRNA biomarkers associated with LNM of CRC that can effectively predict CRC prognosis have not been reported yet.

**Methods**: To identify biomarkers that are associated with LNM, we collected data from the The Cancer Genome Atlas (TCGA) database. The edgeR package was searched to seek LNM-related genes by comparisons between cancer samples and normal colorectal tissues and between LNM and non-LNM (NLNM) of CRC. Univariate and multivariate regression analysis of genes in the intersection to build gene signature associated with independent prognosis of CRC, and then verified by Kaplan-Meier curve and log-rank test, receiver operating characteristic (ROC) curve was used to determine the efficiency of survival prediction of our four-mRNA signature. Finally, the potential molecular mechanisms and properties of these gene signature were also explored with functional and pathway enrichment analysis.

**Results**: 329 mRNAs were up-regulated in CRC tissues with LNM, and 8461 mRNAs were up-regulated in CRC tissues, the intersection is 100 mRNAs. After univariate and multivariate Cox regression analysis of 100 mRNAs, a novel four LNM related mRNAs (EPHA8, KRT85, GABRA3, and CLPSL1) were screened as independent prognostic indicators of CRC. Surprisingly, the four-mRNA signature can predict the prognosis of CRC patients independently of clinical factors andthe area under the curve (AUC) of the ROC is 0.730. The novel four-mRNA signature was used to identify high and low-risk groups. Stratified analysis indicated the risk score based on four-mRNA signature was an independent prognostic indicator for female, T3+T4, N1+N2 ,stage III+IV and patients with no new tumor event. Functional annotation of this risk model in high-risk patients revealed that pathways associated with neuroactive ligand-receptor interaction, estrogen signaling pathway, and steroid hormone biosynthesis.

**Conclusions**: By conducting TCGA data mining, our study demonstrated that a four-mRNA signature associated with LNM can be used as a combined biomarker for independent prognosis of CRC.

## Background

Colorectal cancer (CRC) is one of the common malignant tumors of the digestive tract 1, which seriously threatens the life and quality of life of patients. In both sexes combined, the incidence of CRC is the third (10.2% of the total cases), and the mortality rate is the second (9.2% of the total cancer deaths) 2. At present, the effective treatment of CRC is surgical resection 3, but it is easy to relapse and metastasis after surgery. The most common metastatic sites of CRC are liver, abdominal lymph nodes and lung metastases. According to reports, 5-year survival rate in the United States of CRC is close to 70%, but due to the presence of lymph node metastasis (LNM) or distant metastases, 5-year survival rate under the same conditions fell to 13% 4.The LNM severely affects the survival of CRC patients^5^, so the exploration of biomarkers with high sensitivity and specificity for diagnosis of CRC associate with LNM has become a key problem in the medical field.

Currently researches on CRC-related biomarkers continue to grow 6, for example, related studies have shown that vascular endothelial growth factor (VEGF) is expressed in about 50% of CRC, which is very rarely expressed in normal colon mucosa and adenoma, and VEGF-1 can effectively predict the prognosis of CRC patients 7. PTTG1 (Pituitary tumor transforming gene-1) is an independent prognostic factor that affects the proliferation, invasion and migration of CRC cells 8. Moreover, Zhang JS et al. found patients with high expression of RABEX-5 mRNA had a poor prognosis, RABEX-5 mRNA may be a potential biomarker for evaluating the prognosis of CRC 9. miRNAs are involved in the development and progression of tumors 10, in recent years, miRNAs have also been found as biomarkers for invasion, metastasis and prognosis of CRC 11. Compared with a single biomarker, the advantage of combining biomarkers is that it can increase the sensitivity of detection. Therefore, in order to improve the sensitivity of clinical diagnosis of tumor biomarkers, we can screen combined biomarkers of CRC. Andrea Angius et al. found an integrated signature of 20 deregulated miRNAs that could be evaluated as potential prognostic biomarkers 12, Chuanpeng Dong et al. identified that an eight-gene signature in cancer stem cell signaling was associated with the overall survival patients with CRC 13. Furthermore, the researcher identified a 6-gene signature predicting prognosis for CRC 14. Moreover, LNM is a common mode of metastasis and an important factor affecting the prognosis of CRC 15, there are very few reports on mRNA combination biomarkers for LNM of CRC, so differentially expressed mRNA associated with LNM should be the key to the progression of CRC, we can screen for mRNA combined biomarkers of CRC from this perspective.

In this work, we analyzed 614 patients with CRC in The Cancer Genome Atlas (TCGA) database and found that 100 mRNAs were up-regulated both in CRC patients and CRC patients with LNM. By further analysis we found that based on four-mRNA signature patients with high risk scores have a poorer prognosis and four-mRNA signature associated with LNM can effectively predict the prognosis of CRC patients.

## Methods and Materials

### Patient and mRNA expression data procession

**Figure 1:**
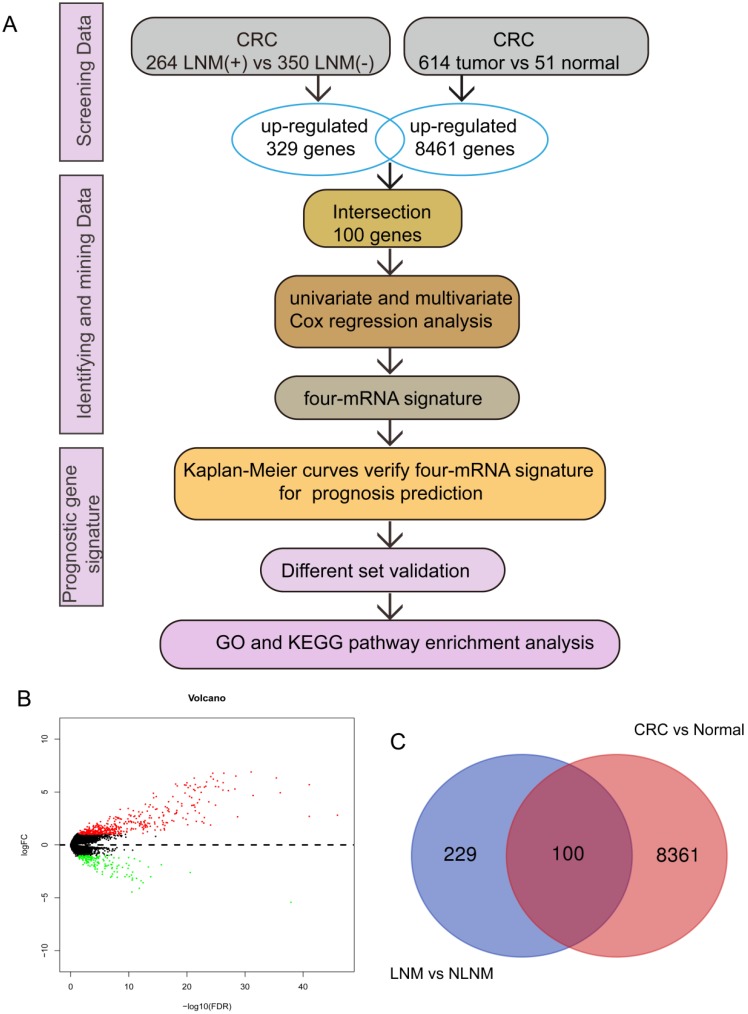
** DEGs associated with LNM.** (A) study design. (B) The volcano map of DEGs in CRC tissues with LNM vs NLNM, the red represents the up-regulated genes. (C) the intersection of the up-regulated genes in LNM vs NLNM tissues and CRC vs non-cancer tissues.

The expression of CRC mRNA and the corresponding clinical data were downloaded from the TCGA database. According to the inclusion criteria: (a) complete gene expression and survival information (b) the CRC patients with LNM were filtered by the criteria that N stage of patients was I-IV, and the exclusive criteria were as follows: (a) survival information or gene expression is incomplete (b) N stage in clinical pathological parameters is not available, we finally obtained a total of 614 CRC tissues and 51 normal colorectal tissues of mRNA expression profiles for further research, among them, 614 tissues included 264 tissues with LNM and 350 tissues with non-LNM (NLNM) (Figure 1A).

### Differential expression of CRC mRNA data mining

Screening for differentially expressed mRNA of 614 CRC tissues and 51 normal colorectal tissues in TCGA using edgeR package, the threshold was set to |logFC| > 1and adjusted *p* value < 0.05, 264 CRC tissues with LNM and 350 CRC tissues with NLNM for differential mRNA mining under the same conditions.

Using Venn digram web-tool (http://bioinformatics.psb.ugent.be/webtools/Venn/) to find the intersection of up-regulated genes in CRC tissues and CRC tissues with LNM.

### Construction of independent prognostic indicators based on mRNA

In our work, 614 CRC patients were randomly divided into two groups (test set N=322, validation set N=292) (Table 1). Then the mRNA expression profile was subjected to log2 transformation for further statistical analysis, and univariate Cox was used to screen mRNAs affecting OS of patients (*p* < 0.05), followed by multivariate Cox regression analysis to identify mRNAs as independent prognostic indicators. Subsequently, based on the expression level of each mRNA and the regression coefficient obtained from multivariate Cox conduct a risk score, risk score =

Exp_mRNA1_×β_mRNA1_+Exp_rnRNA2_×β_mRNA2_+⋯+Exp_mRNAn_×β_mRNAn_

(Exp represents the expression level of each mRNA and β represents the regression coefficient of each mRNA).

### Functional enrichment analysis

In this study, Gene Ontology (GO) and Kyoto Encyclopedia of Genes and Genomes (KEGG) pathway enrichment analysis was performed for these up-regulated mRNAs in patients with high risk score by using the Database for Annotation, Visualization, and Integrated Discovery online tool (https://david.ncifcrf.gov/). GO terms and KEGG pathways with a false discovery rate (FDR) < 0.05 were statistically significant.

### Statistical Analysis

According to the median value of risk scores, 614 CRC patients were divided into high-risk group and low-risk group. Kaplan-Meier curve and log-rank test were used for plotting survival curves. The area under the curve (AUC) of the receiver operating characteristic (ROC) curves used to determine the predicted power of the prognostic gene signature. Moreover, we applied univariate and multivariate Cox analysis to evaluate whether or not the risk score was an independent factor of the other clinical variables including age, T, N, M, stage, residual tumor and neoplasm cancer status in patients with CRC. We used Pearson test or Fisher's exact test to analyze the correlation between LNM and clinical pathological parameters. All statistical analysis was using SPSS 16.0 and GraphPad Prism7.

## Results

### The intersection of differentially expressed genes (DEGs) in CRC

We screened differentially expressed genes (|logFC| > 1, *p* < 0.05) in 614 CRC tissues and 51 normal colorectal tissues and found that 8461 genes were up-regulated in CRC tissues, while 264 CRC tissues with LNM and 350 CRC tissues with NLNM for differential mRNA mining under the same conditions (Figure 1B), then a total of 329 genes were obtained from up-regulated genes in CRC tissues with LNM. In addition, we used the Venn diagram web-tool to cross the two sets of up-regulated genes, as shown in Figure 1C, 100 genes were in the intersection (Supplementary Table S1).

### Identification of four mRNAs associated with prognosis in CRC

**Figure 2:**
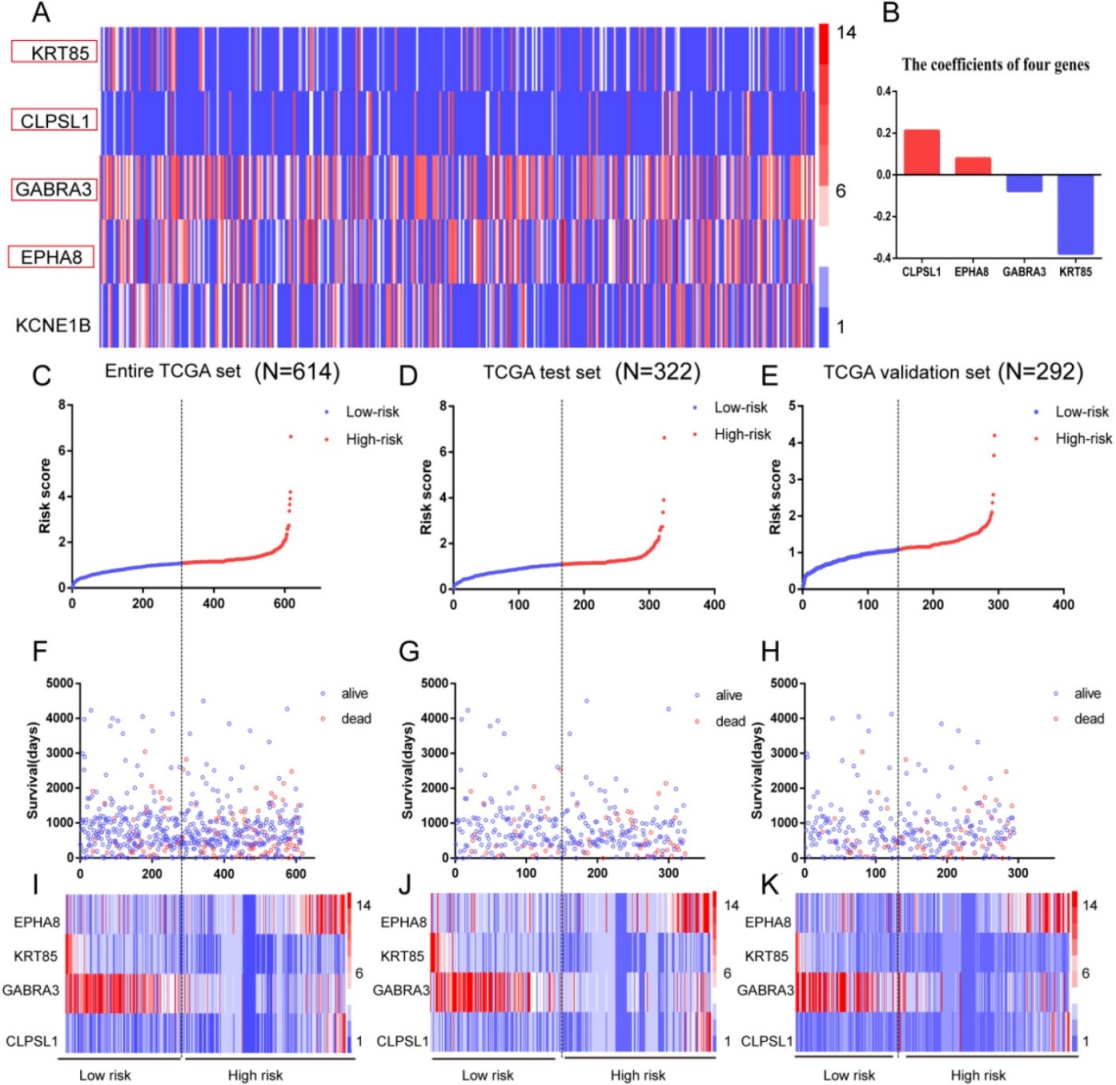
** Risk score analysis of the four-mRNAs signature of CRC.** (A) The heat map of five genes in CRC patients. Each column represents a patient and each row represents a gene. The expression levels of genes are displayed in different colors. From blue to red, the expression is gradually increasing. (B) The coefficients of the four genes, red for positive numbers and blue for negative numbers. (C-E)The distribution of high and low risk scores of four mRNAs in entire TCGA set (N=614), TCGA test set (N=322) and validation set (N=292). (F-H) Survival time and status of patients based on the high and low risk scores of four mRNAs in entire TCGA set (N=614)、TCGA test set (N=322) and validation set (N=292). (I-K) The heat map of four genes in entire TCGA set (N=614), TCGA test set (N=322) and validation set (N=292).

We first used univariate Cox regression analysis to identify 100 intersection genes associated with prognosis, and got five genes with *p* values < 0.05 (Figure 2A). Then multivariate Cox regression analysis was performed and four mRNAs (EPHA8, KRT85, GABRA3, and CLPSL1) were finally screened as prognostic signature models (as shown in Table 2). Among them, EPHA8 and CLPSL1 showed positive coefficients, indicating they are risk factors since their high expression is accompanied by a shorter survival. Instead, we found that KRT85 and GABRA3 are negative coefficients, which means they can be considered protective mRNA and high expression of these mRNAs suggests that patients have longer survival (Figure 2B).

Next, the risk score for predicting OS was established using the formula of the four mRNAs based on the multivariate Cox regression analysis results above: risk score = 0.079 × expression of EPHA8 + 0.212 × expression of CLPSL1-0.377 × expression of KRT85-0.077 × expression of GABRA3. Entire TCGA set (N=614), test set (N=322) and validation set (N=292) respectively were divided into low-risk and high-risk groups according to the median of the prognosis risk score (Figure 2C-E). Next, we analyzed the survival and status of patients in the high and low risk score group, and the results showed that the mortality rate of the high-risk score group is higher than that of the low-risk score group (Figure 2F-H). The heat map results showed that the risk mRNA (EPHA8 and CLPSL1) was up-regulated with increasing risk score and the protective mRNA (KRT85 and GABRA3) expression was down-regulated (Figure 2I-K).

### The four-mRNA signature as a prognostic indicator independent of clinical characteristics

**Figure 3:**
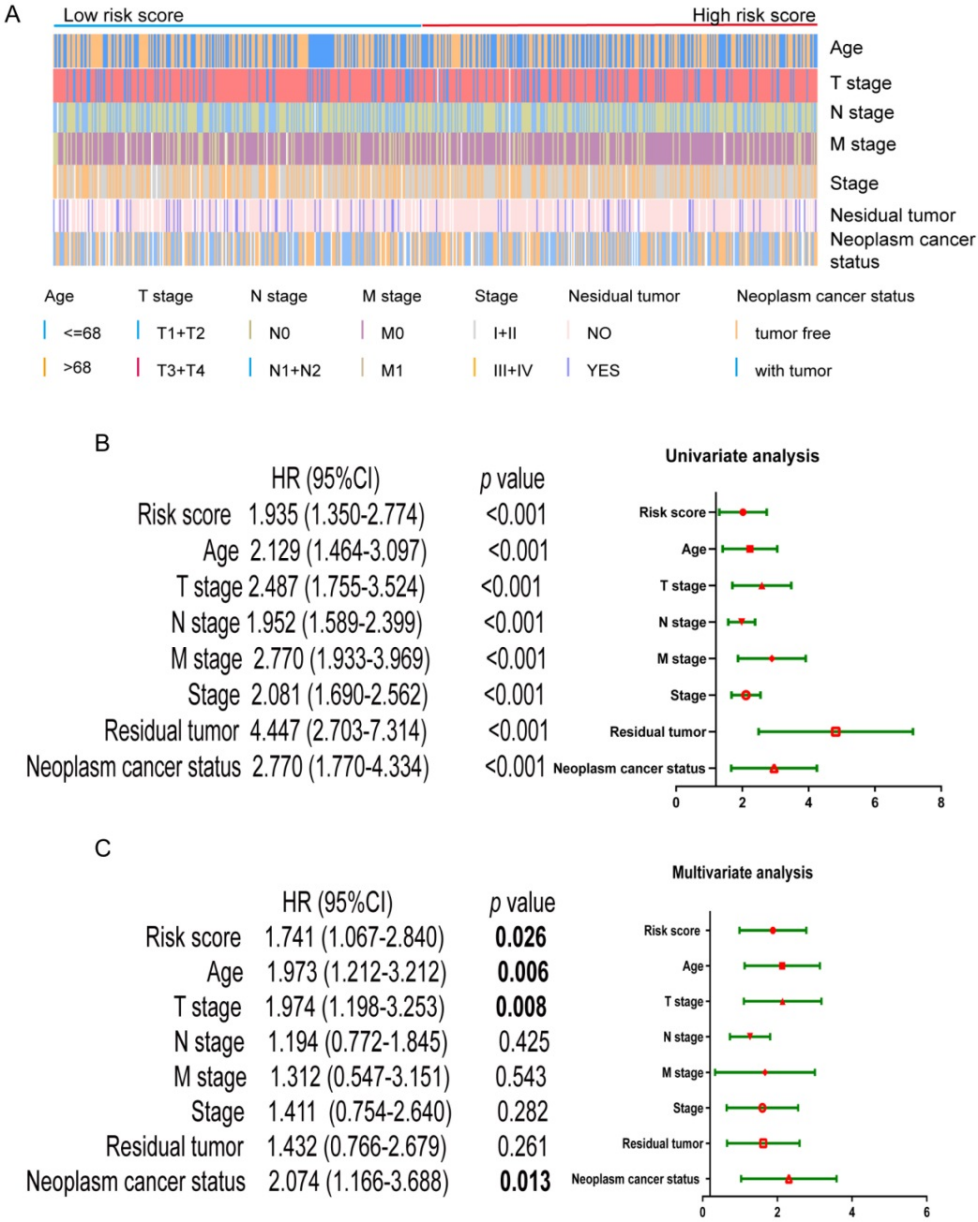
** Univariate and multivariate Cox regression analysis of OS.** (A) Distribution of the clinic pathological parameters including age, T, N, M, stage, residual tumor and neoplasm cancer status in CRC patients with low-risk score to high-risk score. (B) univariate Cox regression analysis of OS. (C) multivariate Cox regression analysis of OS.

First of all, we looked at the distribution of different clinical parameters in patients with low to high risk scores (Figure 3A). Next, the risk score and the clinic pathological parameters including age, T stage, N stage, M stage, stage, residual tumor and neoplasm cancer status were used as explanatory variables, and the OS rate was used as a dependent variable for univariate and multivariate Cox regression analysis. As shown in Figure 3B, univariate Cox regression analysis showed that the four-mRNA risk score and the above mentioned conventional clinic pathological factors can effectively predict the prognosis of patients with CRC.

Among them, "Residual tumor" is the most obvious clinical and pathological parameters predicting the prognosis of patients with CRC, because the probability of death in patients with residual tumors is 4.472 times that of patients without residual tumors. In addition, risk score, age, T stage, and neoplasm cancer status were also significantly different in multivariate analysis (*p* < 0.05), indicating that they can be used as independent prognostic indicators for CRC patients (Figure 3C).

### Kaplan-Meier curves verify four-mRNA signature for survival prediction

**Figure 4:**
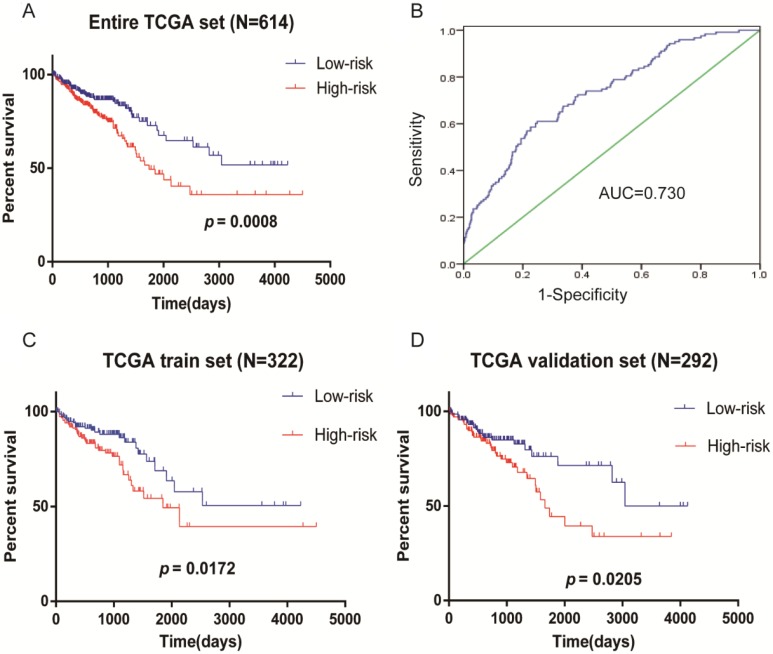
** Kaplan-Meier survival analysis of CRC patients in TCGA data.** (A) Risk score predicts survival of patients with CRC in entire TCGA set (N=614). (B) ROC curves of the four-mRNA signature in CRC (AUC = 0.730). (C) Risk score predicts survival of patients with CRC in TCGA test set (N=322). (D) Risk score predicts survival of patients with CRC in TCGA validation set (N=292).

The Kaplan-Meier curves showed that the prognosis of patients with high-risk scores was poorer (Figure 4A), the AUC of the ROC curves used to determine the predicted power of the prognostic gene signature, the AUC of the four-mRNA signature was 0.730 (Figure 4B). It is also confirmed in test set (N=322) (Figure 4C) and validation set (N=292) (Figure 4D) that patients with high-risk scores had a worse prognosis. These results indicate the four-mRNA signature can effectively predict the prognosis of patients with CRC. Previous univariate Cox regression analysis of OS showed that age, T stage, N stage, M stage, stage, residual tumor and neoplasm cancer status in clinical pathological parameters could effectively predict survival in patients with CRC. Next, we use the Kaplan-Meier method to verify the above conclusion, the results indicate that patients who were older than 68 years, who were in T3+T4, N1+N2, M1, and stage III+IV, who had residual tumors and who had neoplasm cancer have a poorer prognosis(Supplementary Figure S1A). This result further confirms the accuracy of our previous analysis.

**Figure 5:**
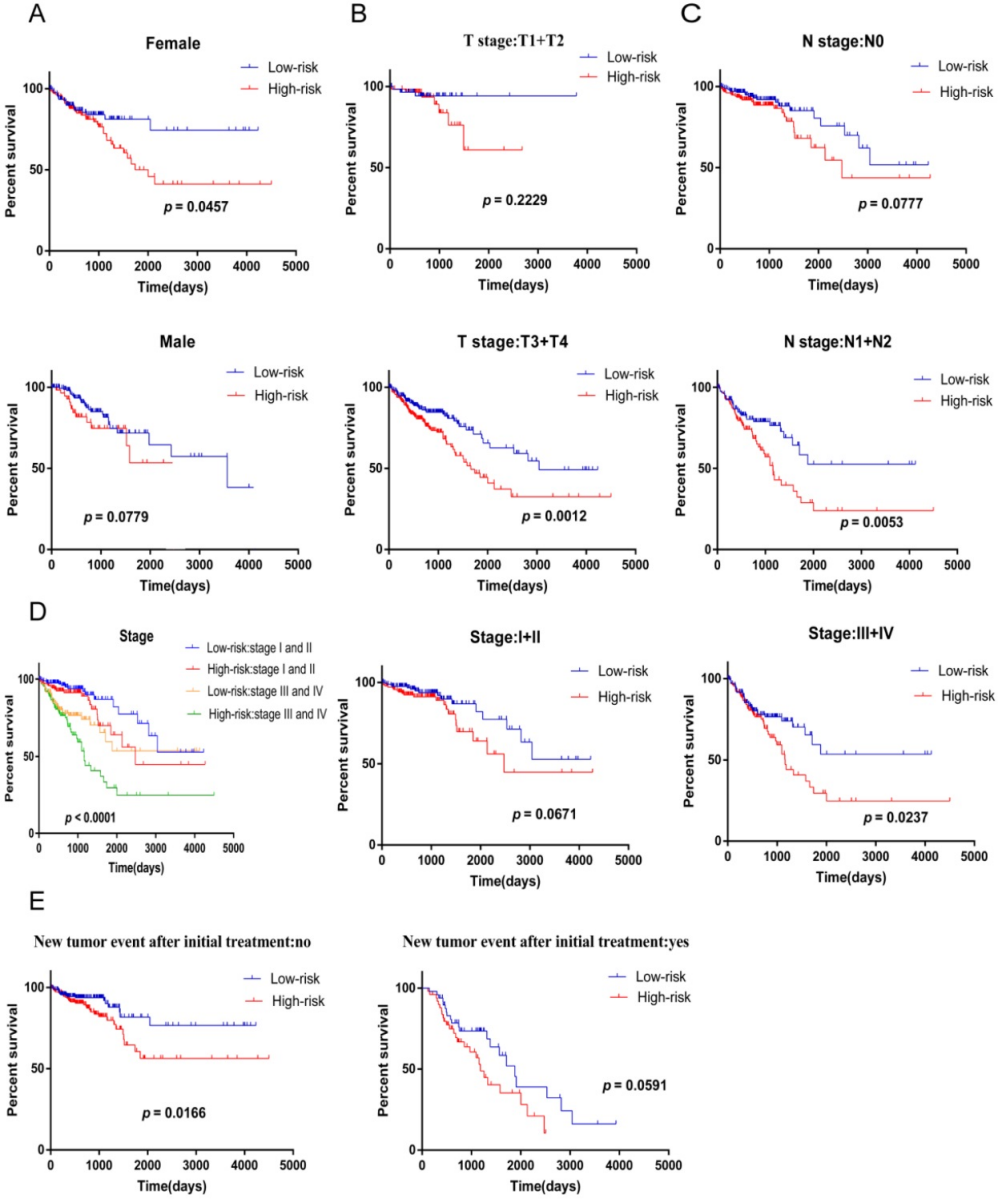
** Kaplan-Meier curves predicts patient survival with clinical features for the patients divided into high and low risk scores.** (A) gender. (B) T stage. (C) N stage. (D) stage.( E) new tumor event after initial treatment.

Next, we used stratified analysis for further data mining and we found that four-mRNA signature is a prognostic marker for female patients with CRC (Figure 5A). After stratification of T stage, N stage, and stage, respectively, the risk score based on four-mRNA signature was an independent prognostic indicator for T3+T4, N1+N2 and stage III+IV, and patients with high risk scores had a poorer prognosis (Figure 5B-D). However, according to the new tumor event after initial treatment, four-mRNA signature was found to be a prognostic marker for patients with no new tumor event, and the high-risk subgroup survived for a shorter period of time (Figure 5E).

### Identification of related potential functions of the four-mRNA signature

**Figure 6:**
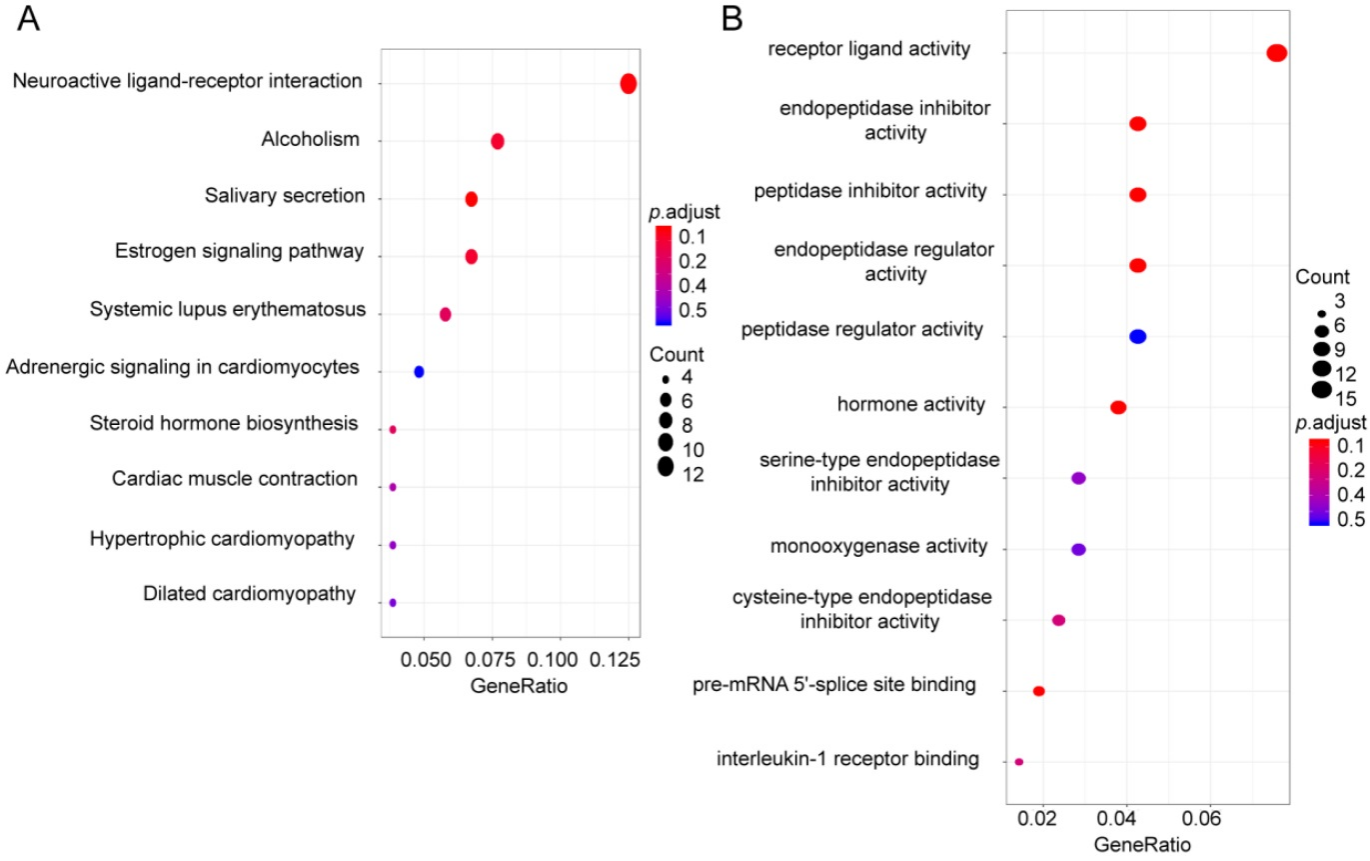
** Functional enrichment analysis**. (A) KEGG analysis of up-regulated genes in high risk score group. (B) GO analysis of up-regulated genes in high risk score group.

To identify pathways and biological processes in which four-mRNA signatures work, we divided 614 CRC patients into low risk group (N=307) and high risk group (N=307) according to the median risk score and screened for differential genes (|logFC| > 1, *p* < 0.05), among them, 112 genes were up-regulated in high risk group (Supplementary Table S2). Next, the GO and KEGG enrichment analysis was performed on these 112 genes. The results showed that these genes were together enriched in pathways in neuroactive ligand-receptor interaction, estrogen signaling pathway, and steroid hormone biosynthesis (Figure 6A) and the results suggested that the top GO biological process were receptor ligand activity, endopeptidase inhibitor activity and peptidase inhibitor activity (Figure 6B).

## Discussion

CRC is a heterogeneous malignant tumor, which makes some clinical parameters such as gender, age, residual tumors, and stage unable to accurately predict the prognosis of CRC patients 16. With the development of high-throughput sequencing, microarray technology and bioinformatics, more and more biomarkers have been discovered to effectively predict the prognosis of patients with CRC 17. For example, Ai-Jun Sun et al. started bioinformatics data mining from the DNA methylation information in the tcga database and found that MSX1 and DCLK1, which are involved in DNA methylation, may be used as biomarkers for CRC 18. Zong Z et al found that MSI-2 is highly expressed in CRC tissues. Surprisingly, logistic regression analysis showed that MSI-2 is associated with liver metastasis of CRC, and MSI-2 is expected to be a biomarker for liver metastasis of CRC 19. Moreover, recent studies have found that TIMP1 is an independent prognostic indicator for disease-free survival and OS in colon cancer patients through the Cox proportional hazards model 20. These findings make mRNAs a promising biomarker for predicting CRC survival. However, in the clinically, a single biomarker is susceptible to various factors, making the combination marker a research hotspot. In recent years, research on combined biomarkers has also been emerging. Zhang Z et al. 21 indicated a set of circRNAs that may serve as a candidate diagnostic biomarker, Dai W et al. 22 revealed an integrated mRNA-lncRNA signature with predictive value of early relapse in colon cancer, Dai W et al. 23 discovered a gene signature for the prediction of early relapse in stage I-III colon cancer and researcher identified a 14-lncRNA prognostic signature for patients with colon adenocarcinoma 24.

It is well known that the treatment of tumors has made great progress in recent years, but metastatic malignant tumors are often incurable, and metastasis is considered to be the main cause of tumor treatment failure 25. Metastasis is a multistep and complex process 26, and the specific mechanism behind it is still not very clear. Tumor metastasis refers to the process in which tumor cells migrate from the primary site through lymphatic vessels or blood vessels to other parts to continue to grow 27. Biomarkers associated with LNM are critical for diagnosis and prognosis of cancer. For example, studies have found that 14-3-3β and profilin-1 can be used to predict LNM of gastric cancer and 14-3-3β may become an independent prognostic marker for gastric cancer 28. Metastasis is an important factor affecting the prognosis and survival of CRC patients 29. Moreover, LNM is a major shift in the form of CRC, the effective removal of LNM of CRC surgery is the focus of a direct impact on the prognosis of patients. Compared with CRC patients without LNM, the OS of patients with LNM was shorter 30. Tsuyoshi Ozawa et al. discovered a 5-miRNA signature which associated with LNM of CRC by tcga RNA sequence mining 31. A recent study showed that SATB1 is highly expressed in the CRC of LNM and may be used as a biomarker for CRC 15. In this study, we analyzed the correlation of LNM and clinic parameters, and the results showed that LNM was associated with T stage, M stage, stage, new tumor event after initial treatment, lymphatic invasion and residual tumor (*p* < 0.05, Supplementary Table 3).Therefore, the identification of genes associated with LNM can be indispensable for the diagnosis and treatment of CRC.

In this research, we analyzed 614 CRC patients and their clinical information in the TCGA database. We used the up-regulated genes screened in CRC patients with LNM to intersect with genes up-regulated in CRC tissues. Next, through COX regression analysis, it was found that the four-mRNA signature (EPHA8, CLPSL1, KRT85, and GABRA3) was closely related to the prognosis of patients with CRC. We further compared the four mRNAs expression between patients with LNM and with NLNM. The results showed that EPHA8, KRT85, and GABRA3 and CLPSL1 were highly expressed in patients with LNM (*p* < 0.05, Supplementary Figure S2A-D). Patients were divided into low risk group and high risk group according to the risk score of four-mRNA signature. Surprisingly, patients in high-risk group had worse prognosis, then the training set and validation set are well validated. By further stratification analysis, the risk score based on four-mRNA signature was an independent prognostic indicator for female, T3+T4, N1+N2, stage III+IV and no new tumor event. Although the risk score is not statistically significant in male, T1+T2, N0, stage I+II and new tumor event, we can also find that the high risk score group has a poorer survival. The result suggests that four-mRNA signature have the potential to be used as a combined biomarker for CRC prognosis. The GO and KEGG enrichment analysis shows that genes up-regulated in the high risk score group are mainly associated with receptor ligand activity, neuroactive ligand-receptor interactions and estrogen signaling pathway. Neuroactive ligand-receptor interactions have been found to be associated with multiple cancers 32, estrogen signaling pathway plays an important role in the development of CRC 33, receptor ligand activity affects invasion and metastasis of CRC. This result provides new insights and research ideas for the four-mRNA signatures affecting the prognosis of patients with CRC.

EphA8 is one of the receptors in Ephs subfamily of receptor tyrosine kinases, it is associated with angiogenesis, cell adhesion and migration. Overexpression of EphA8 enhances the invasive ability of oral squamous cell carcinoma 34, miR-10a/EphA8 pathway can affect glioma invasion and migration through epithelial-mesenchymal transition 35. In our study, the HR of EPHA8 > 1, this means that patients with high expression of EphA8 have a poorer prognosis. Gammaaminobutyric acid (GABA) is an inhibitory neurotransmitter, and Gabra3 is a subunit of GABA type A receptor 36. In previous research, Gumireddy K et al. found that GABRA3 can promote cell invasion, migration and metastasis through AKT pathway in breast cancer 37, Recent studies have shown that miR-92b-3p targets and reduces GABRA3 expression and thus inhibits pancreatic cancer cell invasion and migration 38, GABRA3 also plays an important role in the occurrence and development of liver cancer and lung cancer 39, 40. However, the role of GABRA3 in CRC is still unclear. Interestingly, in this work, GABRA3 is a protective factor, but the role of GABRA3 in CRC needs further research.

For the first time, our study reported a four-mRNA signature associated with LNM for prognosis of CRC using bioinformatics methods. In this work, patients with high risk scores have a poorer prognosis. Independent of other clinic pathological parameters, four-mRNA signature may become a combined biomarker for predicting the prognosis of CRC patients. However, the four-mRNA signature should be validated in a larger sample size database as well as in clinical samples. Moreover, our results warrant further studies of the mechanisms by which the four-mRNA signature affects prognosis of CRC.

## Conclusions

The results of this study indicate that a four-mRNA signature related with LNM can effectively predict the prognosis of patients with CRC, but the specific molecular mechanism of the four-mRNA acting on colorectal cancer and whether this four-mRNA signature can be successfully applied to the clinic still need further research.

## Supplementary Material

Supplementary figures and tables.

## Figures and Tables

**Table 1:** Clinical characteristic of patients with CRC in each set.

Clinical feature	Test set(%)	Validation set(%)	Entire set (%)
**Gender**			
Male	178(55.3)	148(50.7)	326(53.1)
Female	144(44.7)	144(49.3)	288(46.9)
**Age**			
< = 68	166(51.6)	162(55.5)	328(53.4)
> 68	156(48.4)	130(44.5)	286(46.6)
**T stage**			
T1+T2	61(19.0)	64(21.9)	125(20.4)
T3+T4	260(80.7)	228(78.1)	488(79.5)
unknown	1(0.3)	0(0.0)	1(0.1)
**N stage**			
N0	194(60.2)	156(53.4)	350(57.0)
N1+N2	128(39.8)	136(46.6)	264(43.0)
**M stage**			
M0	235(73.0)	222(76.0)	457(74.4)
M1	46(14.3)	41(14.0)	87(14.2)
unknown	41(12.7)	29(10.0)	70(11.4)
**Stage**			
I+II	181(56.2)	150(51.4)	331(53.9)
III+IV	131(40.7)	136(46.6)	267(43.5)
unknown	10(3.1)	6(2.0)	16(2.6)
**Neoplasm cancer status (with tumor/tumor free)**			
Tumor free	135(41.9)	120(41.1)	255(41.5)
With tumor	152(47.2)	137(46.9)	289(47.1)
unknown	35(10.9)	35(12.0)	70(11.4)
**New tumor event after initial treatment(yes/no)**			
No	222(68.9)	187(64.0)	409(66.6)
Yes	52(16.1)	47(16.1)	99(16.1)
unknown	48(15.0)	58(19.9)	106(17.3)
**Lymphatic invasion(yes/no)**			
No	171(53.1)	157(53.8)	328(53.4)
Yes	115(35.7)	112(38.4)	227(37.0)
unknown	36(11.2)	23(7.8)	59(9.6)
**Residual tumor(yes/no)**			
No	230(71.4)	219(75.0)	449(73.2)
Yes	22(6.8)	20(6.8)	42(6.8)
unknown	70(31.8)	53(18.2)	123(20.0)

**Table 2:** Details of four prognostic mRNAs significantly associated with OS in CRC.

mRNA	Ensemble ID	Location	β	HR	*p*
EPHA8	ENSG00000070886	chr1: 22563489-22603595	0.079	1.083	0.045
KRT85	ENSG00000135443	chr12:52360006-52367481	-0.377	0.686	0.014
GABRA3	ENSG00000011677	chrX:152166234-152451359	-0.077	0.926	0.036
CLPSL1	ENSG00000204140	chr6:35781017-35794039	0.212	1.236	0.016

## Abbreviations

CRC: colorectal cancer
LNM: lymph node metastasis
NLNM: no lymph node metastasis
DEGs: differentially expressed genes
EPHA8: EPH receptor A8
KRT85: keratin 85
GABRA3: gamma-aminobutyric acid (GABA) A receptor, subunit alpha 3
CLPSL1: colipase like 1
