# Supplementary material for: A Combined four-mRNA Signature Associated with Lymphatic Metastasis for Prognosis of Colorectal Cancer
Source: J Cancer. 2020 Feb 3;11(8):2139–49. doi: 10.7150/jca.38796 (PMC7052913; doi:10.7150/jca.38796)
Supplement: Supplementary file 1 — Supplementary figures and tables. [file jcav11p2139s1.pdf]

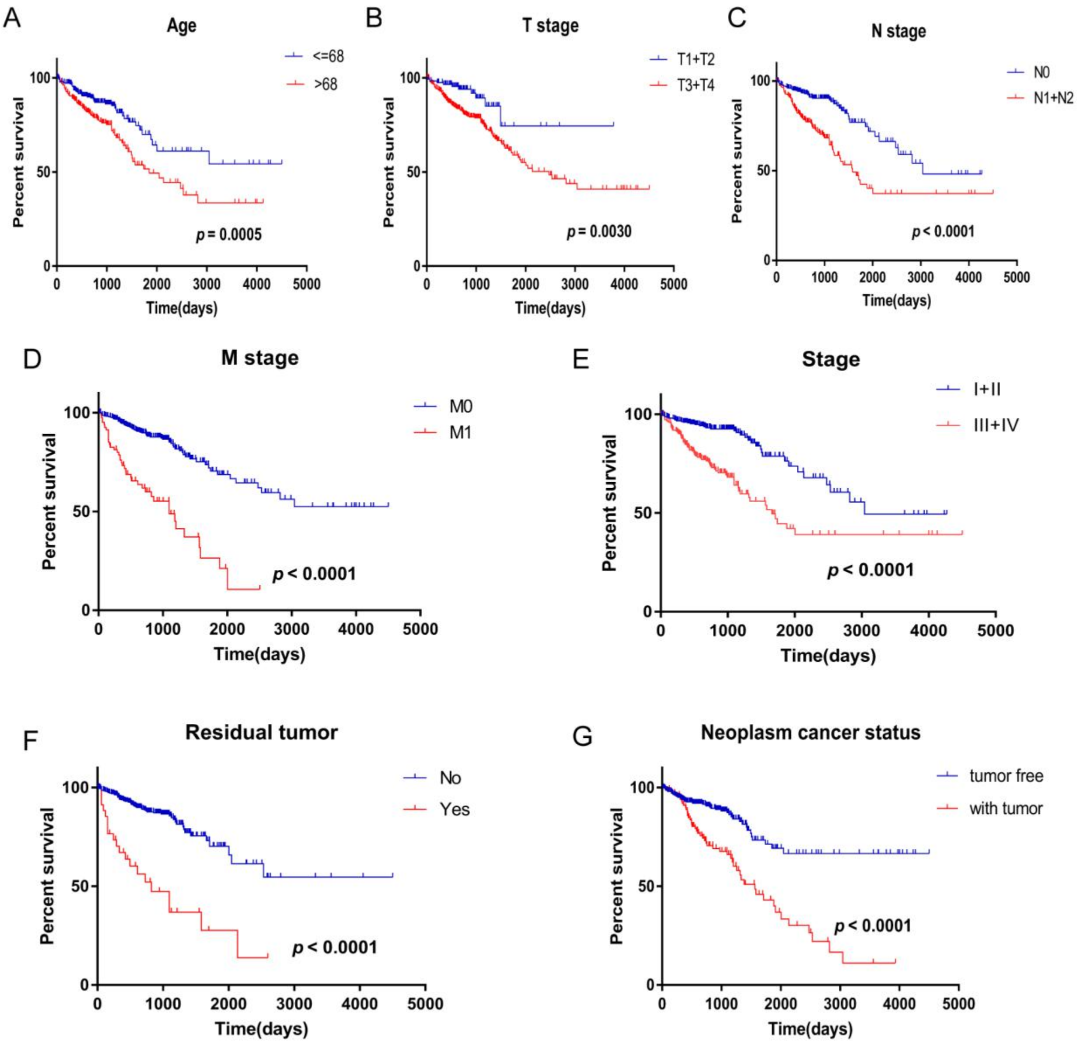

Supplementary Figure S1

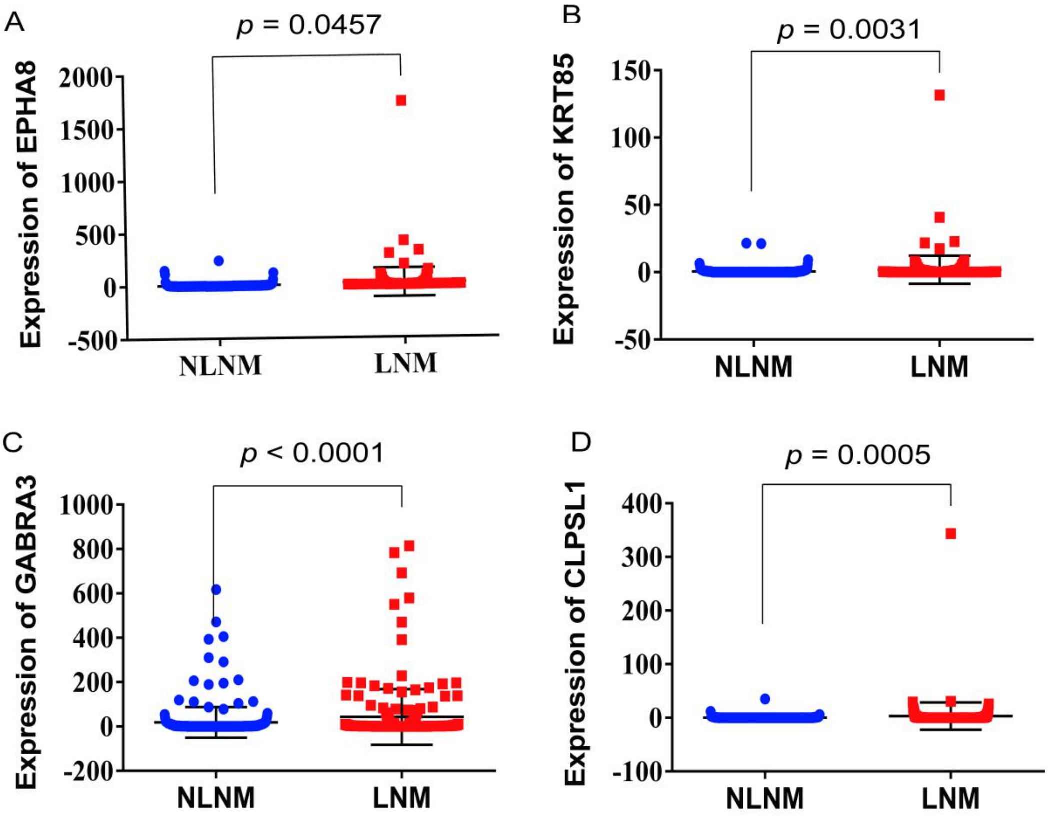

Supplementary Figure S2

Table S1 Information on 100 intersection genes

Gene	Ensemble ID	Location
C6orf15	ENSG00000229432	chr6:31111223-31112559
NFE4	ENSG00000230257	chr7:102973430-102988856
PPBP	ENSG00000163736	chr4:73986439-73988190
UNCX	ENSG00000164853	chr7:1232872-1237326
BRDT	ENSG00000137948	chr1:91949371-92014426
FBXO47	ENSG00000204952	chr17:38936432-38967402
BTBD18	ENSG00000233436	chr11:57743514-57751781
LYPD2	ENSG00000197353	chr8:142750150-142752534
KCNC2	ENSG00000166006	chr12:75040077-75209868
HOXC13	ENSG00000123364	chr12:53938831-53946544
COL20A1	ENSG00000101203	chr20:63293186-63334851
CACNA1E	ENSG00000198216	chr1:181317690-181808084
CXorf67	ENSG00000187690	chrX:51406948-51408843
BAGE2	ENSG00000187172	chr21:10413477-10516431
VCX	ENSG00000182583	chrX:7842262-7844143
PSG9	ENSG00000183668	chr19:43211791-43269530
HRH3	ENSG00000101180	chr20:62214960-62220278
TGM6	ENSG00000166948	chr20:2380908-2432753
ISM2	ENSG00000100593	chr14:77474394-77498816
OR51B5	ENSG00000167355	chr11:5303444-5505652
UPK1A	ENSG00000105668	chr19:35666516-35678483
DDX53	ENSG00000184735	chrX:22999961-23003589
CRP	ENSG00000132693	chr1:159712289-159714589
RDH8	ENSG00000080511	chr19:10013249-10022279
CASP14	ENSG00000105141	chr19:15049480-15058293
CXCL17	ENSG00000189377	chr19:42428288-42443048
PAGE1	ENSG00000068985	chrX:49687447-49695984
CHRNA6	ENSG00000147434	chr8:42752620-42796392
NPVF	ENSG00000105954	chr7:25224570-25228486
KRT84	ENSG00000161849	chr12:52377812-52385652
EPHA8	ENSG00000070886	chr1:22563489-22603595
ZFP42	ENSG00000179059	chr4:187995771-188005046
MAGEB2	ENSG00000099399	chrX:30215563-30220089
TCHH	ENSG00000159450	chr1:152106317-152115454

KRT39	ENSG00000262164	chr17:40958417-40966892
TEX19	ENSG00000182459	chr17:82359247-82363775
PNMT	ENSG00000141744	chr17:39667981-39670475
TMCO2	ENSG00000188800	chr1:40245947-40251684
TUBA3C	ENSG00000198033	chr13:19173772-19181824
APOA5	ENSG00000110243	chr11:116789367-116792420
XAGE2	ENSG00000155622	chrX:52369021-52375680
REG3G	ENSG00000143954	chr2:79025686-79028505
DWORF	ENSG00000240045	chr3:155290227-155293683
COL2A1	ENSG00000139219	chr12:47972967-48004554
NEUROD4	ENSG00000123307	chr12:55019974-55030017
SBK2	ENSG00000187550	chr19:55529733-55537089
KRT85	ENSG00000135443	chr12:52360006-52367481
TBXT	ENSG00000164458	chr6:166157656-166168700
ORM1	ENSG00000229314	chr9:114323056-114326475
CLDN6	ENSG00000184697	chr16:3014712-3020071
TUBA3E	ENSG00000152086	chr2:130191745-130198439
GLYATL2	ENSG00000156689	chr11:58834065-58904215
SYNGR4	ENSG00000105467	chr19:48364395-48376376
WIF1	ENSG00000156076	chr12:65050626-65121305
PAGE2	ENSG00000234068	chrX:55089008-55092842
KCNE1B	ENSG00000276289	chr21:7816675-7829926
CRHR1	ENSG00000278232	chr17:45784280-45835828
MMP8	ENSG00000118113	chr11:102711796-102727050
IRX4	ENSG00000113430	chr5:1877413-1887236
DCAF4L2	ENSG00000176566	chr8:87870747-87874015
SOHLH1	ENSG00000165643	chr9:135693407-135699528
CACNG1	ENSG00000108878	chr17:67044554-67056797
COL26A1	ENSG00000160963	chr7:101362875-101559024
SLURP1	ENSG00000126233	chr8:142740949-142742406
CSNK1A1L	ENSG00000180138	chr13:37103259-37105664
HTR2C	ENSG00000147246	chrX:114584078-114910061
NTF4	ENSG00000225950	chr19:49061066-49065054
ACTL8	ENSG00000117148	chr1:17755333-17827063
TTR	ENSG00000118271	chr18:31557010-31599021
HOXC12	ENSG00000123407	chr12:53954903-53958956
KRTAP3-1	ENSG00000212901	chr17:41,008,521-41,019,324

LHX3	ENSG00000107187	chr9:136,196,250-136,205,128
COLEC10	ENSG00000184374	chr8:118995452-119108455
TMEM213	ENSG00000214128	chr7:138797952-138838101
FGF3	ENSG00000186895	chr11:69809968-69819416
CT45A1	ENSG00000268940	chrX:135713453-135723539
HS3ST4	ENSG00000182601	chr16:25691959-26137685
GABRA3	ENSG00000011677	chrX:152166234-152451359
KRT40	ENSG00000204889	chr17:40977716-40987135
AIRE	ENSG00000160224	chr21:44285838-44298648
UPK2	ENSG00000110375	chr11:118925164-118958559
CER1	ENSG00000147869	chr9:14719724-14722717
GREB1L	ENSG00000141449	chr18:21242242-21525417
INSL6	ENSG00000120210	chr9:5123880-5185647
CALML5	ENSG00000178372	chr10:5498697-5499555
LRP2	ENSG00000081479	chr2:169127109-169362534
KRTAP3-3	ENSG00000212899	chr17:40993430-40994133
KRTAP4-6	ENSG00000198090	chr17:41139433-41140487
FGG	ENSG00000171557	chr4:154604134-154612967
PNMA5	ENSG00000198883	chrX:152988824-152994116
COX7B2	ENSG00000170516	chr4:46734827-46909245
CALCA	ENSG00000110680	chr11:14966668-14972354
VWA5B1	ENSG00000158816	chr1:20290919-20354894
LGALS14	ENSG00000006659	chr19:39704481-39709444
ERVW-1	ENSG00000242950	chr7:92468380-92477986
CDH9	ENSG00000113100	chr5:26880597-27121150
CLPSL1	ENSG00000204140	chr6:35781017-35794039
SSX1	ENSG00000126752	chrX:48255317-48267444
HIST1H4F	ENSG00000274618	chr6:26240426-26240737
SALL3	ENSG00000256463	chr18:78980275-79002677

Table S2 Information on 112 up-regulated genes

Gene	Ensemble ID	Location
PAEP	ENSG00000122133	chr9:135561627-135566776
CALCB	ENSG00000175868	chr11:15073600-15078631
ADAD2	ENSG00000140955	chr16:84191117-84197166
SFTPC	ENSG00000168484	chr8:22161671-22164479
GRM1	ENSG00000152822	chr6:146027634-146437598
FBXO47	ENSG00000204952	chr17:38936278-38967476
KRT78	ENSG00000170423	chr12:52837804-52848994
ZNF479	ENSG00000185177	chr7:57119574-57139899
LRP1B	ENSG00000168702	chr2:140231423-142132463
MMP8	ENSG00000118113	chr11:102711795-102724967
TUBA3E	ENSG00000152086	chr2:130191745-130198461
IRGM	ENSG00000237693	chr5:150846523-150902402
SERPINB4	ENSG00000206073	chr18:63637259-63644319
KRT14	ENSG00000186847	chr17:41582279-41586895
SPRR2E	ENSG00000203785	chr1:153093135-153094528
SERPINB13	ENSG00000197641	chr18:63586654-63599199
SFTPA1	ENSG00000122852	chr10:79610939-79615443
GALP	ENSG00000197487	chr19:56176020-56185775
CRCT1	ENSG00000169509	chr1:152514502-152516010
COX8C	ENSG00000187581	chr14:93347191-93348354
LCE3D	ENSG00000163202	chr1:152579384-152580504
DHRS2	ENSG00000100867	chr14:23630115-23645639
HYPM	ENSG00000187516	chrx:37990817-37991317
PSG9	ENSG00000183668	chr19:43253282-43269563
KRT84	ENSG00000161849	chr12:52377665-52386977
CHRNA2	ENSG00000120903	chr8:27459761-27479296
TP63	ENSG00000073282	chr3:189596746-189897279
SPRR2A	ENSG00000241794	chr1:153056120-153057512
PNLDC1	ENSG00000146453	chr6:159799422-159820704
CALML5	ENSG00000178372	chr10:5498695-5499570
TDRD12	ENSG00000173809	chr19:32719773-32829580
TMCO2	ENSG00000188800	chr1:40247901-40251693
KCNC2	ENSG00000166006	chr12:75040078-75209748
CLEC2A	ENSG00000188393	chr12:9882733-9932430
S100A7	ENSG00000143556	chr1:153457744-153460661
FAM41C	ENSG00000230368	chr1:868071-876802
SPRR2D	ENSG00000163216	chr1:153039725-153041144
KRT13	ENSG00000171401	chr17:41500981-41505613
SERPINB3	ENSG00000057149	chr18:63655197-63661963
NTRK3	ENSG00000140538	chr15:87859749-88256796
UTS2B	ENSG00000188958	chr3:191266465-191330536
SPRR3	ENSG00000163209	chr1:153001747-153003856

KRT6C	ENSG00000170465	chr12:52468516-52473785
SMC1B	ENSG00000077935	chr22:45344063-45413671
TMPRSS11E	ENSG00000087128	chr4:68447449-68497604
CBLN4	ENSG00000054803	chr20:55997357-56005472
TMEM229A	ENSG00000234224	chr7:124030916-124033469
KRT6A	ENSG00000205420	chr12:52487174-52493397
NFE4	ENSG00000230257	chr7:102973437-102978667
KRT16	ENSG00000186832	chr17:41609778-41612827
CCDC160	ENSG00000203952	chrx:134237213-134246207
NR0B1	ENSG00000169297	chrx:30304422-30309378
CTAG2	ENSG00000126890	chrx:154651972-154653579
ERVW-1	ENSG00000242950	chr7:92468380-92477915
CLCA2	ENSG00000137975	chr1:86423883-86456558
CLDN6	ENSG00000184697	chr16:3014712-3018187
IL36G	ENSG00000136688	chr2:112978019-112985672
MAJIN	ENSG00000168070	chr11:64938230-64972098
ACTL8	ENSG00000117148	chr1:17755313-17827063
SBSN	ENSG00000189001	chr19:35523367-35528371
RPE65	ENSG00000116745	chr1:68428822-68450322
CYP17A1	ENSG00000148795	chr10:102830531-102837533
IL36RN	ENSG00000136695	chr2:113058638-113064744
IGFL1	ENSG00000188293	chr19:46229752-46231243
PINCR	ENSG00000224294	chrx:43176994-43226598
XAGE2	ENSG00000155622	chrx:52368996-52375683
MUC15	ENSG00000169550	chr11:26559032-26572268
S100G	ENSG00000169906	chrx:16649787-16654674
SLC35F4	ENSG00000151812	chr14:57563921-57982327
TMPRSS11D	ENSG00000153802	chr4:67820876-67885063
ITLN2	ENSG00000158764	chr1:160945020-160957378
KRT40	ENSG00000204889	chr17:40977716-40987135
IGDCC3	ENSG00000174498	chr15:65327127-65378040
A2ML1	ENSG00000166535	chr12:8822472-8887202
SSX1	ENSG00000126752	chrx:48255317-48267444
IVL	ENSG00000163207	chr1:152908545-152911886
HTN1	ENSG00000126550	chr4:70050415-70058848
DDX53	ENSG00000184735	chrx:22999961-23002089
SALL3	ENSG00000256463	chr18:78980275-78998969
MAGEC2	ENSG00000046774	chrx:142202342-142205290
PDYN	ENSG00000101327	chr20:1978756-1994285
SFTPB	ENSG00000168878	chr2：85657317-85668741
REG1B	ENSG00000168878	chr2:79085023-79088024
SERPINB2	ENSG00000197632	chr18:63887705-63903890
UPK1A	ENSG00000105668	chr19:35666516-35678485
TCF23	ENSG00000163792	chr2:27148892-27153586

LMX1A	ENSG00000162761	chr1:165201867-165356715
SULT1E1	ENSG00000109193	chr4:69841212-69860152
FAM133A	ENSG00000179083	chrx:93673716-93712274
PCA3	ENSG00000225937	chr9:76764436-76787569
SPRR1B	ENSG00000169469	chr1:153031203-153032900
TRHDE-AS1	ENSG00000236333	chr12:72253507-72273509
BRINP3	ENSG00000162670	chr1:190097662-190477997
CYP7A1	ENSG00000167910	chr8:58490178-58500161
LYPD2	ENSG00000197353	chr8:142750150-142752534
CCDC190	ENSG00000185860	chr1:162851291-162868918
COL2A1	ENSG00000139219	chr12:47972965-48006212
PRB1	ENSG00000251655	chr12:11351823-11355590,
OR2T10	ENSG00000184022	chr1:248589668-248645472
CHST9	ENSG00000154080	chr18:26908754-27185364
PRSS56	ENSG00000237412	chr2:232520463-232525716
SEMG2	ENSG00000124157	chr20:45221369-45224458
CLPSL1	ENSG00000204140	chr6:35781017-35794039
IL36B	ENSG00000136696	chr2:113022091-113052867
MAGEC1	ENSG00000155495	chrx:141903856-141909401
CSAG1	ENSG00000198930	chrx:152727484-152733736
DSCR8	ENSG00000198054	chr21:38121451-38156511
MAGEA10	ENSG00000124260	chrx:152133310-152138578
KRT24	ENSG00000167916	chr17:40694246-40703750
MAGEA12	ENSG00000213401	chrx:152733779-152737669
SAGE1	ENSG00000181433	chrx:135893607-135913061
CIDEA	ENSG00000176194	chr18:12254319-12277595

Table S3: Correlation between LNM and clinicopathological features of CRC patients

Clinical feature	LNM(%)	NLNM(%)	*p*
Gender			0.496
Male	136(41.72)	190(58.28)	
Female	128(44.44)	160(55.56)	
Age			0.073
< = 68	152(46.34)	176(53.66)	
> 68	112(39.16)	174(60.84)	
T stage			**<0.001**
T1+T2	16(12.80)	109(87.20)	
T3+T4	248(50.82)	240(49.18)	
M stage			**<0.001**
M0	151(33.04)	306(66.96)	
M1	77(88.51)	10(11.49)	
Stage			**<0.001**
I+II	0(0.00)	331(100.00)	
III+IV	257(96.25)	10(3.75)	
Neoplasm cancer status (with tumor/tumor free)			0.178
Tumor free	101(39.61)	154(60.39)	
With tumor	131(45.33)	158(54.67)	
New tumor event after initial treatment(yes/no)			**0.001**
No	164(40.10)	245(59.90)	
Yes	58(58.59)	41(41.41)	
Lymphatic invasion(yes/no)			**<0.001**
No	80(24.39)	248(75.61)	
Yes	159(70.04)	68(29.96)	
Residual tumor(yes/no)			**<0.001**
No	172(38.31)	277(61.69)	
Yes	36(85.71)	6(14.29)	
